# Anti-Inflammatory Effects of Psoralen Derivatives on RAW264.7 Cells via Regulation of the NF-κB and MAPK Signaling Pathways

**DOI:** 10.3390/ijms23105813

**Published:** 2022-05-22

**Authors:** Yeji Lee, Chang-Gu Hyun

**Affiliations:** Jeju Inside Agency and Cosmetic Science Center, Department of Chemistry and Cosmetics, Jeju National University, Jeju 63243, Korea; cosmetics_chemistry@naver.com

**Keywords:** 5-hydroxypsoralen, 5-methoxypsoralen, 8-hydroxypsoralen, 8-methoxypsoralen, xanthotoxol, NF-κB, MAPK

## Abstract

Using repositioning to find new indications for existing functional substances has become a global target of research. The objective of this study is to investigate the anti-inflammatory potential of psoralen derivatives (5-hydroxypsoralen, 5-methoxypsoralen, 8-hydroxypsoralen, and 8-methoxypsoralen) in macrophages cells. The results indicated that most psoralen derivatives exhibited significantly inhibited prostaglandin E_2_ (PGE_2_) production, particularly for 8-hydroxypsoralen (xanthotoxol) in lipopolysaccharide (LPS)-stimulated macrophage RAW 264.7 cells. In addition, xanthotoxol treatment decreased the PGE_2_, IL-6, and IL-1β production caused by LPS stimulation in a concentration-dependent manner. Moreover, Western blot results showed that the protein levels of inducible nitric oxide synthase (iNOS) and cyclooxygenase-2 (COX-2), which activated with LPS treatment, were decreased by xanthotoxol treatment. Mechanistic studies revealed that xanthotoxol also suppressed LPS-stimulated phosphorylation of the inhibitor of κBα (IκBα), p38 mitogen-activated protein kinase (MAPK), and c-Jun N-terminal kinase (JNK) in RAW 264.7 cells. The Western blot assay results show that xanthotoxol suppresses LPS-induced p65 translocation from cytosol to the nucleus in RAW 264.7 cells. Moreover, we tested the potential application of xanthotoxol as a cosmetic material by performing human skin patch tests. In these tests, xanthotoxol did not induce any adverse reactions at a 100 μΜ concentration. These results demonstrate that xanthotoxol is a potential therapeutic agent for topical application that inhibits inflammation via the MAPK and NF-κB pathways.

## 1. Introduction

Most small molecule drugs interact with one target protein or more. Drug discovery—via drug repositioning, one of the most popular and successful strategies—is being promoted under the polypharmacology premise. Various in vitro assays and in silico methods, including omics- and molecular-docking-based methods, have been used to perform systematic assessments of the potential for existing drugs to be used outside of their original medical indication [1,2,3,4,5,6].

Our screening process to discover existing drugs with the potential to be used outside of their current scope included skin inflammation and melanogenesis drugs which deviated from the original medical indication. We observed that several antibiotics have novel functionaries on inflammation and melanogenesis. These results suggest that tobramycin and fosfomycin enhanced melanogenesis via MAPK signaling pathways in B16F10 melanoma cells [7,8]. In addition, it was reported that spiramycin, cycloserine, and nojirimycin attenuate inflammation via NF-κB and MAPK signaling pathways in LPS-induced RAW 264.7 macrophages [9,10,11]. Furthermore, we have identified that spiramycin and cycloserine inhibited melanogenesis via MAPK/PKA/AKT signaling pathways in α-MSH-treated mouse melanoma B16F10 cells (unpublished data).

Although this strategy is similar to drug repurposing in terms of drug discovery, the repositioning of natural products is also garnering attention [12]. While most drug repurposing campaigns rely on compounds derived from chemical synthesis, natural products offer significant opportunities due to their unique and beneficial properties, significant structural diversity, and large number of pharmacological activities [13]. Therefore, interest in developing new drugs from natural products is once again growing; however, the way this phenomenon is developed currently differs significantly from the past. Recently, this method has been accelerating the “reuse” of nature-inspired compounds [14,15].

During our ongoing screening program, designed to reuse natural compounds, we reported that several flavonoids and coumarins have anti-inflammatory, adipogenesis-inhibitory, and melanogenic activities applicable to cosmeceuticals and nutraceuticals. As an extension of this study, we screened several psoralen derivatives, which have a similar, simple structure, to identify the structural features involved in the bioactivities of this class of molecules [16,17,18,19,20]. 

Psoralen is a furocoumarin natural product which consists of a coumarin moiety-fused furan ring [21]. Psoralen and its derivatives are known for clinical efficacy in several skin diseases, including cutaneous lichen planus, graft-versus-host disease, mycosis fungoides, vitiligo, and psoriasis [22,23]. Given the therapeutic relevance of psoralen and its derivatives, many studies aim to synthesize psoralens with even greater potency [24,25]. 

**Figure 1:**
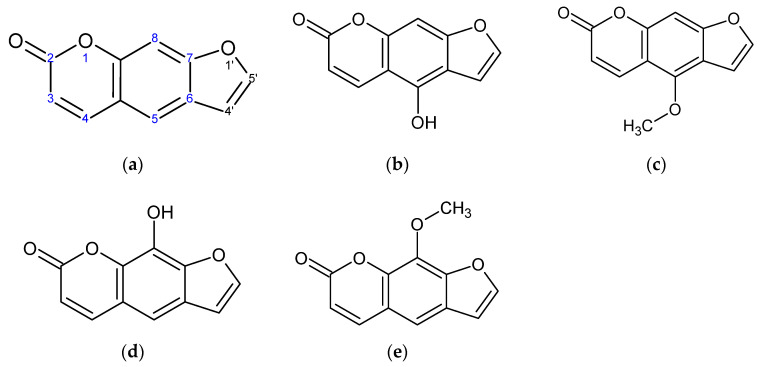
Structure of psoralen derivatives. (**a**) Psoralen, (**b**) 5-hydroxypsoralen (bergaptol), (**c**) 5-methoxypsoralen (bergapten), (**d**) 8-hydroxypsoralen (xanthotoxol), and (**e**) 8-methoxypsoralen (xanthotoxin).

In this study, we observed that, among the compounds screened, four psoralen derivatives—5-hydroxypsoralen (bergaptol), 5-methoxypsoralen (bergapten), 8-hydroxypsoralen (xanthotoxol), and 8-methoxypsoralen (xanthotoxin), which have similar and simple structures (Figure 1)—exhibit distinct potency which is derived from their structural differences. In addition, the anti-inflammatory potential of each of the psoralen derivatives was evaluated based on their capacity to reduce NO and PGE_2_ levels in LPS-induced RAW 264.7 macrophages, selected due to it being the most promising psoralen derivative. Then, a mechanistic study was conducted.

## 2. Results

### 2.1. Effect of Psoralen Derivatives on the Viability of RAW 264.7 Cells

**Figure 2:**
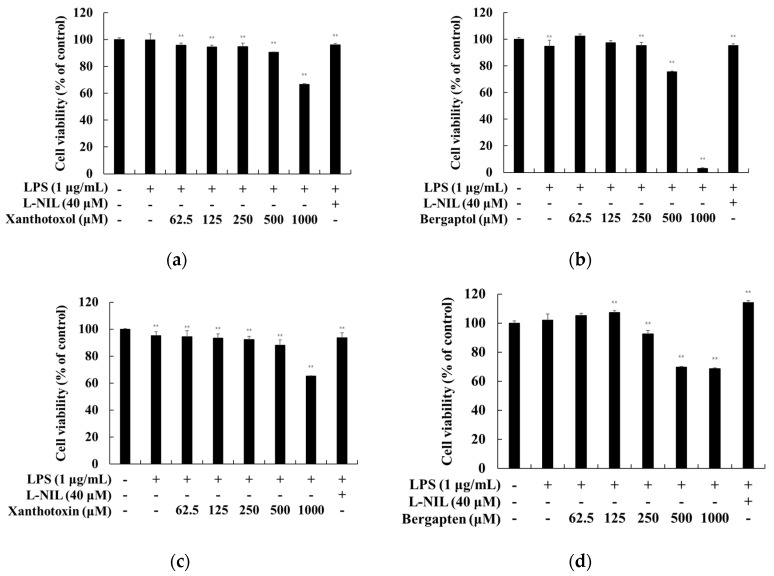
Effect of psoralen derivatives on the cell viability in LPS-induced RAW 264.7 cells. The cells were treated with psoralen derivatives (62.5, 125, 250, 500, and 1000 μM) and L-NIL (40 μM) for 24 h with LPS (1 μg/mL). L-NIL was used as a positive control. The cell viability of LPS-induced RAW 264.7 cells subjected to (**a**) 8-hydroxypsoralen (xanthotoxol), (**b**) 5-hydroxypsoralen (bergaptol), (**c**) 8-methoxypsoralen (xanthotoxin), and (**d**) 5-methoxypsoralen (bergapten) were measured using an MTT assay. The results are presented as the mean ± SD from three independent experiments. ** *p* < 0.01 vs. untreated control group.

To investigate whether psoralen derivatives exerted cytotoxicity on RAW 264.7 cells, the cells were treated with various concentrations (62.5, 125, 250, 500, and 1000 μM) of psoralen derivatives with LPS (1 μg/mL) for 24 h. The results showed that there were no significant differences up to a 250 μM concentration of psoralen derivatives in RAW 264.7 cells (Figure 2). Therefore, we used psoralen derivative concentrations of 62.5, 125, and 250 μM for further experiments.

### 2.2. Effect of Psoralen Derivatives on NO Production of RAW 264.7 Cells

**Figure 3:**
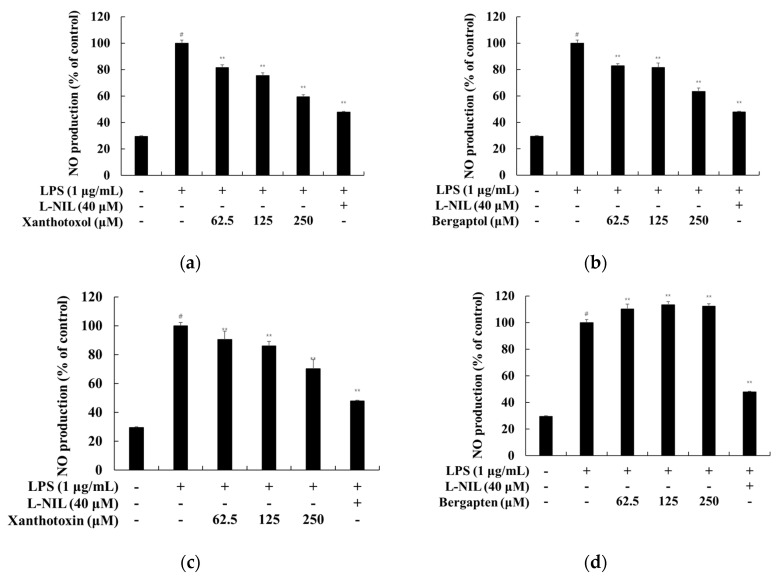
Effect of psoralen derivatives on nitric oxide production in LPS-induced RAW 264.7 cells. The cells were treated with psoralen derivatives (62.5, 125, and 250 μM) and L-NIL (40 μM) for 24 h with LPS (1 μg/mL). L-NIL was used as a positive control. NO production of LPS-induced RAW 264.7 cells subjected to (**a**) 8-hydroxypsoralen (xanthotoxol), (**b**) 5-hydroxypsoralen (bergaptol), (**c**) 8-methoxypsoralen (xanthotoxin), and (**d**) 5-methoxypsoralen (bergapten) treatment were measured using Griess reagents. The results are presented as the mean ± SD from three independent experiments. # *p* < 0.01 vs. untreated control group. ** *p* < 0.01 vs. LPS alone.

To evaluate the NO production of psoralen derivatives on RAW 264.7 cells, the cells were treated with psoralen derivatives (62.5, 125, and 250 μM) and L-NIL (40 μM) with LPS (1 μg/mL) for 24 h. As shown in Figure 3, the generation of NO increased remarkably in response to stimulation with LPS when compared with the control group. However, NO production decreased significantly with psoralen derivative treatments (bergaptol, xanthotoxol, and xanthotoxin). Among these treatments, the inhibitory effect of NO production by xanthotoxol was greater than that of other psoralen derivatives. Therefore, further experiments were performed to evaluate the anti-inflammatory effects of xanthotoxol.

### 2.3. Effect of Xanthotoxol on PGE_2_ and Inflammatory Cytokines

**Figure 4:**
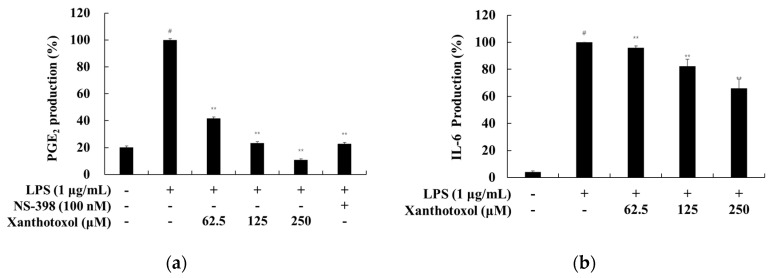

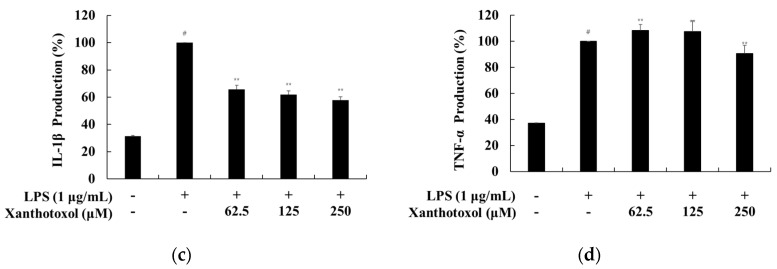
Effect of xanthotoxol on the production of PGE_2_ and inflammatory cytokines in LPS-induced RAW 264.7 cells. The cells were treated with xanthotoxol (62.5, 125, and 250 μM) and LPS (1 μg/mL) for 24 h. NS-398 was used as a positive control. (**a**) PGE_2_, (**b**) IL-6, (**c**) IL-1β, and (**d**) TNF-α production were determined by ELISA kit. The results are presented as the mean ± SD from three independent experiments. # *p* < 0.01 vs. untreated control group. ** *p* < 0.01 vs. LPS alone.

We investigated whether xanthotoxol inhibits PGE_2_ and inflammatory cytokines (IL-6, IL-1β and TNF-α) in LPS-stimulated RAW 264.7 cells. Our results showed that xanthotoxol inhibited PGE_2_, IL-6, and IL-1β production in a concentration-dependent manner. When compared with the LPS-only treatment group at 250 μM, the amount of PGE_2_ production reduced by 93.24%; when compared with the positive control NS-398, xanthotoxol inhibited production more (Figure 4). However, xanthotoxol pretreatment had no significant effect on TNF-α production compared to the control group in this assay.

### 2.4. Effect of Xanthotoxol on INOS and COX-2 Production

**Figure 5:**
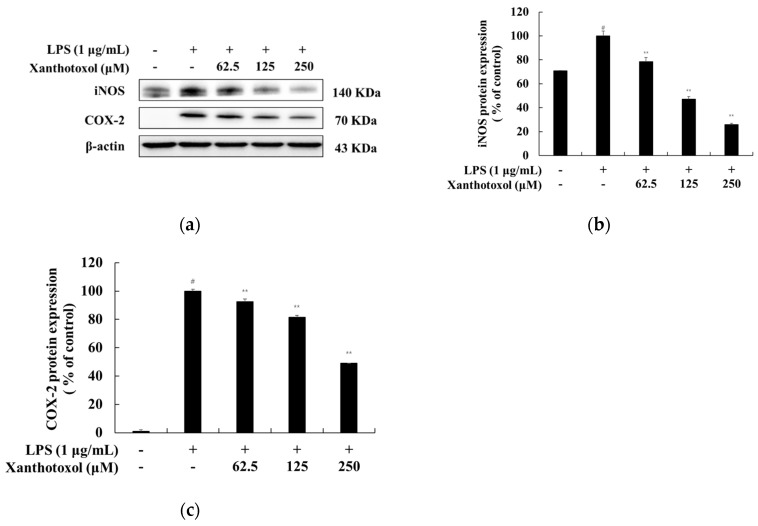
Effect of xanthotoxol on the protein expression level of iNOS and COX-2 in LPS-induced RAW 264.7 cells. The cells were treated with xanthotoxol (62.5, 125, and 250 μM) and LPS (1 μg/mL) for 24 h. (**a**) Western blotting results, and protein expression of (**b**) iNOS and (**c**) COX-2. β-actin was used as a loading control. The results are presented as the mean ± SD from three independent measurements using the Image J. # *p* < 0.01 vs. untreated control group. ** *p* < 0.01 vs. LPS alone.

We investigated whether the inhibition of NO and PGE_2_ production by xanthotoxol in RAW 264.7 cells stimulated with LPS was due to the downregulation of iNOS and COX-2 production inhibition. This was investigated by performing a Western blot experiment. The results showed that xanthotoxol inhibited iNOS and COX-2 production in a concentration-dependent manner. Therefore, xanthotoxol reduced LPS-induced NO and PGE_2_ by suppressing the expression of iNOS and COX-2 (Figure 5).

### 2.5. Effect of Xanthotoxol on the MAPK Signaling Pathway

**Figure 6:**
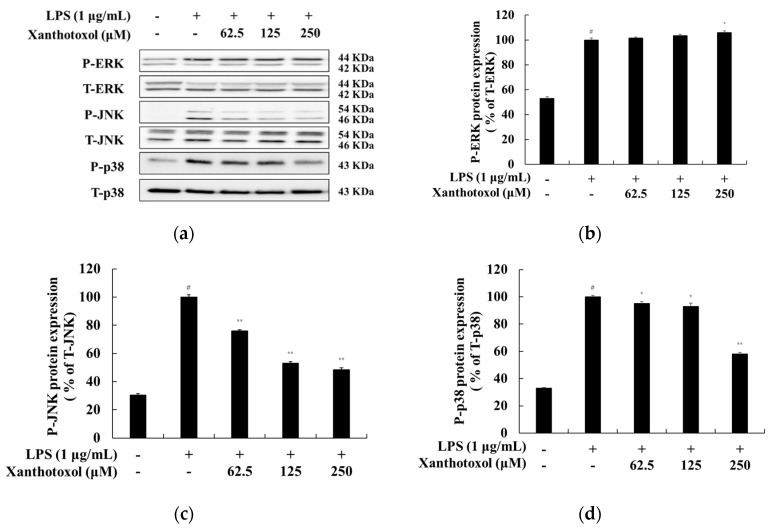
Effect of xanthotoxol on phosphorylation level of MAPK in LPS-induced RAW 264.7 cells. The cells were treated with xanthotoxol (62.5, 125, and 250 μM) and LPS (1 μg/mL) for 20 min. (**a**) Western blotting results, and protein expression of (**b**) P-ERK/T-ERK, (**c**) P-JNK/T-JNK, and (**d**) P-p38/T-p38. β-actin was used as a loading control. The results are presented as the mean ± SD from three independent measurements using the Image J. # *p* < 0.01 vs. untreated control group. * *p* < 0.05 and ** *p* < 0.01 vs. LPS alone.

It has been reported that the phosphorylation of MAPK activates signaling pathways and increases the production of various inflammatory cytokines. Therefore, to investigate and confirm whether xanthotoxol inhibits NO and production of inflammatory cytokines through the MAPK signaling pathway in LPS-stimulated RAW 264.7 cells, a Western blot experiment was conducted. The results show that xanthotoxol inhibited LPS-induced phosphorylation of JNK and p38. This suggests that xanthotoxol regulates inflammation through the JNK and p38 signaling pathways (Figure 6).

### 2.6. Effect of Xanthotoxol on the NF-κB Signaling Pathway

**Figure 7:**
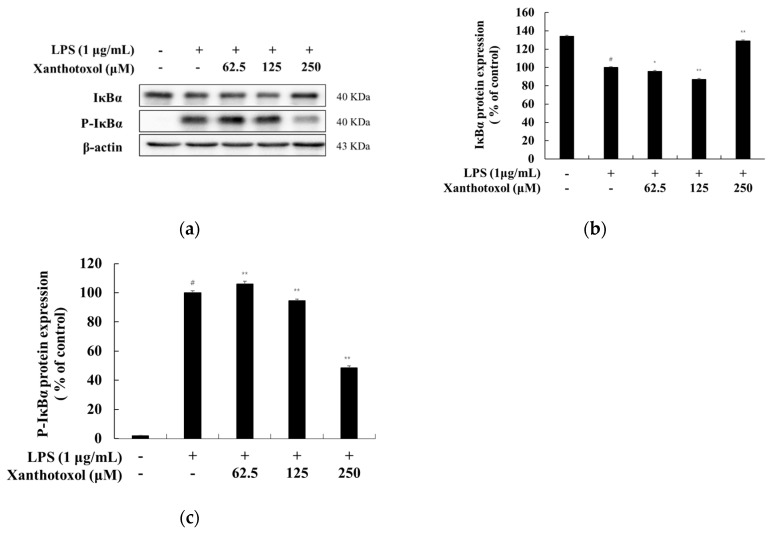
Effect of xanthotoxol on protein expression level of P-IκBα and IκBα in LPS-induced RAW 264.7 cells. The cells were treated with xanthotoxol (62.5, 125, and 250 μM) and LPS (1 μg/mL) for 20 min. (**a**) Western blotting results, and protein expression of (**b**) IκBα and (**c**) P-IκBα. β-actin was used as a loading control. The results are presented as the mean ± SD from three independent measurements using the Image J. # *p* < 0.01 vs. untreated control group. * *p* < 0.05 and ** *p* < 0.01 vs. LPS alone.

**Figure 8:**
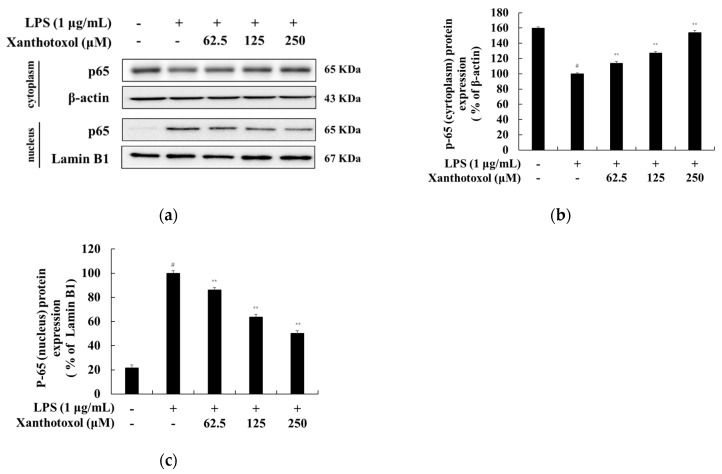
Effect of xanthotoxol on protein expression level of NF-κB (p65) in LPS-induced RAW 264.7 cells. The cells were treated with xanthotoxol (62.5, 125, and 250 μM) and LPS (1 μg/mL) for 15 min. (**a**) Western blotting results; protein expression of (**b**) p65 (cytoplasm) and (**c**) p65 (nuclear). β-actin and Lamin B1 were used as a loading control. The results are presented as the mean ± SD from three independent measurements using the Image J. # *p* < 0.01 vs. untreated control group. ** *p* < 0.01 vs. LPS alone.

It has been reported that, when the macrophage is stimulated with LPS, IκBα is phosphorylated and ubiquitinated. Accordingly, phosphorylated NF-κB is translocated from the cytoplasm to the nucleus to increase the inflammatory cytokine [9,10,11]. Western blot experiments were performed to investigate whether xanthotoxol inhibits the production of inflammatory cytokines through the NF-κB signaling pathway in LPS-stimulated RAW 264.7 cells. The results show that xanthotoxol increased IκBα expression induced by LPS treatment and decreased phosphorylated IκBα induced by LPS treatment (Figure 7). Next, we confirmed the translocation of NF-kB (p65) from the cytoplasm to the nucleus. In the cytoplasm, p65 expression was reduced in cells stimulated by LPS. However, it was confirmed that xanthotoxol increased p65 expression in a concentration-dependent manner. In the nucleus, p65 expression was increased in cells stimulated by LPS, but xanthotoxol reduced p65 expression in a concentration-dependent manner. These results indicate that xanthotoxol inhibits inflammation by preventing IκBα degradation and the nuclear translocation of NF-κB (Figure 8).

### 2.7. Skin Primary Irritation Test

Xanthotoxol at a concentration of 100 µM was applied to a patch and tested for 24 h with skin contact. Then, the area was observed 48 h after the patch was removed. As shown in Table 1, the test substance (xanthotoxol) was classified in the “none to slight” category. Squalene was used as a negative control.

## 3. Discussion

The strategy of developing new ingredients applicable to human health by verifying the newfound efficacy of existing natural products has become a topic of global interest and is comparable to drug repurposing [12,13]. Madecasic acid and asiaticoside, ingredients for wound healing ointment derived from *Centella asiatica*, have become global raw materials applied in wrinkle-improving cosmetics [26,27]. In addition, magnolol and honokiol, antibacterial substances derived from *Magnolia kobus*, are topical applications for acne skin diseases [28,29]. 

In the process of screening strategies for existing natural products, our researchers reported various research results, such as the whitening and antiobesity effects of pinostilbene [16,17], the whitening effect of acanthotic acid [20], and the improvement of skin diseases in methyl jasmonate [19]. In this study, we tried to verify the new efficacy of psorlene, and first applied it to anti-inflammatory or skin diseases using four psoralene derivatives. In the present study, we showed the inhibitory effects of psoralen derivatives (5-hydroxypsoralen, 5-methoxypsoralen, 8-hydroxypsoralen, and 8-methoxypsoralen) on inflammatory pathogenesis, based on their capacity to reduce cellular NO and PGE_2_ levels. In addition, 8-hydroxypsoralen (xanthotoxol) exhibited more potent effects against PGE_2_ production in LPS-induced RAW 264.7 macrophages than 5-hydroxypsoralen, 5-methoxypsoralen, and 8-methoxypsoralen. The present study showed that xanthotoxol alleviated inflammation through suppressing the secretions of NO, IL-6, and TNF-α and expressions of iNOS and COX-2 in LPS-induced RAW 264.7 cells. These results were consistent with the previous study, which reported that xanthotoxol has an anti-inflammatory effect [30]. However, this earlier study only excludes the role of NO; the underlying molecular mechanisms remain to be explored. Of note, we were the first to suggest that xanthotoxol exhibits anti-inflammatory activity. Our results strongly suggest that xanthotoxol has protective effects against inflammation in LPS-treated macrophages. 

The inhibition of NF-κB and MAPKs signaling has been used as the important indicator for the development of anti-inflammatory drugs [10,19]. We examined the underlying mechanism, MAPKs and NF-κB signaling pathways, modulating inflammatory responses in RAW 264.7 cells. Here, we found that xanthotoxol reduced the phosphorylation levels of JNK and p38 of the MAPK family, participating in numerous pathological processes such as inflammation, cell apoptosis, gene transcription, and differentiation. In addition, xanthotoxol attenuated the phosphorylation levels of IκBα and inhibited the nuclear translocation of NF-κB p65 in the NF-κB pathway. These results indicated that the anti-inflammatory effects of xanthotoxol are at least partially mediated by inhibiting MAPK and NF-κB activation. Contradictions in the expressions of IκBα in LPS-induced macrophages were found in previous studies. In some studies, LPS treatment increased overall protein expression and inhibited phosphorylation, whereas other studies showed only increased phosphorylation in IκBα without increasing protein expression. These different results may be caused by differences in the processing time and/or antibody source [31,32]. Our present results showed that LPS-treatment-induced phosphorylation of IκBα was reversed by xanthotoxol at a concentration of 250 μM, while also increasing the expression of total IκBα.

In conclusion, our data reveal that xanthotoxol exhibits anti-inflammatory activity that is dependent on its ability to regulate the production of NO, PGE_2_, and other cytokines in LPS-induced RAW 264.7 cells through the suppression of NF-κB activation and MAPKs phosphorylation. Moreover, xanthotoxol did not induce any severe adverse reactions in the human skin irritation tests. Considering these results, we suggest that xanthotoxol could be considered as a possible anti-inflammatory candidate for topical application. Further research is required to determine its anti-inflammatory properties against skin diseases such as acne and atopic dermatitis.

## 4. Materials and Methods

### 4.1. Chemicals and Reagents

Xanthotoxol (8-hydroxypsoralen), bergaptol (5-hydroxypsoralen), and bergapten (5-methoxypsoralen) were purchased from ChemFaces (Wuhan, China), and xanthotoxin (8-methoxypsoralen) was purchased from TCI (Tokyo, Japan). Lipopolysaccharide from *Escherichia coli* (LPS), Griess reagent, protease inhibitor cocktail, α-melanocyte stimulating hormone (α-MSH), sodium hydroxide (NaOH), and L-dopa were purchased from Sigma-Aldrich (St. Louis, MO, USA). 3-(4,5-dimethylthiazol-2-yl)-2,5-diphenyltetrazolium bromide (MTT) and dimethyl sulfoxide (DMSO) were purchased from Biosesang (Seongnam, Gyeonggi-do, Korea). Dulbecco’s Modified Eagle’s Medium (DMEM), penicillin–streptomycin, and NE-PER nuclear and cytoplasmic extraction reagents were purchased from Thermo Fisher Scientific (Waltham, MA, USA). Fetal bovine serum (FBS) was purchased from Merck Millipore (Burlington, MA, USA). PGE_2_ was purchased from Abcam (Cambridge, CB2 0AX, UK), and TNF-α, IL-1β, IL-6 were purchased from BD Biosciences (Franklin Lakes, NJ, USA). The primary antibodies used for Western blot, p-ERK, ERK, p-JNK, JNK, p-p38, p38, p-IκB-α, IκB-α, β-actin, p65, and Lamin B, were purchased from Cell Signaling Technology (Danvers, MA, USA). Anti-iNOS antibody was purchased from Merck Millipore (Burlington, MA, USA) and Anti-COX-2 antibody was purchased from BD Biosciences (Franklin Lakes, CA, USA). All the reagents used were of analytical grade.

### 4.2. Cell Culture

RAW 264.7 murine macrophage cells were purchased from Korean Cell Line Bank. The cells were cultured in DMEM supplemented with 1% penicillin/streptomycin and 10% FBS at 37 °C under a humidified incubator of 5% CO_2_. RAW 264.7 cells were subcultured every 2 days.

### 4.3. Cell Viability

Cell viability was measured using the MTT assay. RAW 264.7 cells were seeded in a 24-well plate at 1.5 × 10^5^ cells/well and incubated for 24 h. Next, the cells were treated with various concentrations of samples (62.5, 125, 250, 500, and 1000 μM) for 24 h. The medium was removed and DMSO was added into each well to dissolve purple formazan crystals, and absorbance was measured at 570 nm using a spectrophotometric microplate reader.

### 4.4. Nitric Oxide

The NO production was measured in the form of nitrite in the cell culture medium using Griess reagent. RAW 264.7 cells were seeded in a 24-well plate at 1.5 × 10^5^ cells/well for 24 h. The cells were treated with various concentrations of samples (15.6, 31.3, 62.5, 125, and 250 μM) with LPS (1 μg/mL) for 24 h. Then, 100 μL of the supernatant and 100 μL of Griess reagent were mixed in a 96-well plate. Absorbance was measured at 540 nm using a microplate reader. L-NIL (40 μM), the inhibitor of inducible nitric oxide synthase, was used as positive control.

### 4.5. PGE_2_ and Cytokines 

PGE_2_ and cytokines (IL-6, IL-1β, and TNF-α) were measured using an ELISA Kit. RAW 264.7 cells were seeded in a 24-well plate at 1.5 × 10^5^ cells/well for 24 h. The cells were treated with various concentrations (62.5, 125, and 250 μM) of xanthotoxol with LPS (1 μg/mL) for 24 h. NS-398, a COX-2 inhibitor (100 nM), was used as a positive control in the PGE_2_ production experiment. The supernatant was obtained from each well, and the levels of PGE_2_, IL-6, IL-1β, and TNF-α were measured according to the manufacturer’s protocols of the ELISA kit.

### 4.6. Preparation of Nuclear and Cytoplasmic Extraction

Nuclear and cytoplasmic extracts were isolated using an extraction reagent kit. RAW 264.7 cells were seeded in 60 mm cell culture dishes at 6.0 × 10^5^ cells for 24 h. The cells were treated with various concentrations (62.5, 125, and 250 μM) of xanthotoxol with LPS (1 μg/mL) and incubated according to each protein expression time. After incubation, a nuclear extract was obtained according to the manufacturer’s protocols for the extraction reagent kit.

### 4.7. Western Blotting

RAW 264.7 cells were seeded in 60 mm cell culture dishes at 6.0 × 10^5^ cells for 24 h. The cells were treated with xanthotoxol (62.5, 125, and 250 μM) and LPS (1 μg/mL) for each protein expression time. After incubation, they were washed with 1× PBS buffer and lysed using lysis buffer at 4 °C for 10 min. Then, the cells were scraped with a cell scraper and transferred to a 1.5 mL e-tube. After vortexing three times at 10 min intervals, lysates were centrifuged at 15,000 rpm, −8 °C for 20 min to obtain supernatants. The protein level was quantified using a BCA protein assay kit, and heated at 100 °C for 5 min. Then, 20 μg of protein in each sample was loaded on 10% (*v*/*v*) sodium dodecyl sulfate-polyacrylamide gel (SDS-PAGE). The proteins were separated by size by electrophoresis. Proteins were transferred to a polyvinylidene fluoride (PVDF) membrane. The membrane was blocked with 5% (*w*/*v*) skimmed milk for 1 h and washed with 0.1% Tween 20 (TBS-T) for 10 min a total of six times. The membrane was incubated overnight at 4 °C with the primary antibody (1:1000). Then, the membranes were washed with 1× TBS-T and reacted for 2 h at room temperature using a secondary antibody (1:2000). After washing, specific proteins were detected using an ECL kit.

### 4.8. Human Skin Patch Test

Overall, 31 volunteers between 20 and 60 years of age who have never experienced irritation and/or allergic contact dermatitis were included in the study. Their ages ranged from 23 to 51 years, with the average age being 42.03 years. The xanthotoxol formulated with squalane was prepared as the negative control and applied at 100 μM concentrations. The primary skin irritation response was evaluated in accordance with the PCPC guidelines. The skin reaction results for each test substance were calculated from the formula shown below [33,34]. This study was approved by the Institutional Review Board (IRB) of Dermapro Inc. and conducted according to the Declaration of Helsinki as a statement of ethical principles for medical research after obtaining written informed consent from each volunteer (IRB no. 1-220777-A-N-01-DICN20189).

$$\mathrm{Response}=\frac{\sum \left(Grade\times No.\;of\;Responders\right)}{4\;\left(Maximum\;Grade\right)\times n\;\left(Total\;Subjects\right)}\times 100\times 1/2$$

### 4.9. Statistical Analyses

All experiment results were expressed as the mean ± standard deviation (SD) of at least three independent experiments. Statistical analyses were performed using Student’s t-tests or one-way ANOVA using IBM SPSS (v. 20, SPSS Inc., Armonk, NY, USA); *p*-values < 0.05 (*) or 0.01 (**) were marked as statistically significant.

## Figures and Tables

**Table 1:** Results of human skin primary irritation test (*n* = 31).

No.	Test Samples	No. of Responder	24 h				48 h				Reaction Grade		
			+1	+2	+3	+4	+1	+2	+3	+4	24 h	48 h	Mean
1	Xanthotoxol (100 μM)	0	-	-	-	-	-	-	-	-	0	0	0
2	Control (Squalene)	0	-	-	-	-	-	-	-	-	0	0	0

## Data Availability

Not applicable.

## References

[1] Huang B., Zhang Y. (2022). Teaching an old dog new tricks: Drug discovery by repositioning natural products and their derivatives. Drug Discov. Today.

[2] Pushpakom S., Iorio F., Eyers P.A., Escott K.J., Hopper S., Wells A., Doig A., Guilliams T., Latimer J., McNamee C. (2019). Drug repurposing: Progress, challenges and recommendations. Nat. Rev. Drug Discov..

[3] Barreca M., Spanò V., Raimondi M.V., Bivacqua R., Giuffrida S., Montalbano A., Cavalli A., Bertoni F., Barraja P. (2022). GPCR Inhibition in Treating Lymphoma. ACS Med. Chem. Let..

[4] Spanò V., Barreca M., Cilibrasi V., Genovese M., Renda M., Montalbano A., Galietta L.J.V., Barraja P. (2021). Evaluation of Fused Pyrrolothiazole Systems as Correctors of Mutant CFTR Protein. Molecules.

[5] Barreca M., Ingarra A.M., Raimondi M.V., Spanò V., De Franco M., Menilli L., Gandin V., Miolo G., Barraja P., Montalbano A. (2022). Insight on pyrimido[5,4-g]indolizine and pyrimido[4,5-c]pyrrolo[1,2-a]azepine systems as promising photosensitizers on malignant cells. Eur. J. Med. Chem..

[6] Cilibrasi V., Spanò V., Bortolozzi R., Barreca M., Raimondi M.V., Rocca R., Maruca A., Montalbano A., Alcaro S., Ronca R. (2022). Synthesis of 2H-Imidazo[2′,1′:2,3] [1,3]thiazolo[4,5-e]isoindol-8-yl-phenylureas with promising therapeutic features for the treatment of acute myeloid leukemia (AML) with FLT3/ITD mutations. Eur. J. Med. Chem..

[7] Ullah S., Chung Y.C., Hyun C.G. (2020). Induction of Melanogenesis by Fosfomycin in B16F10 Cells Through the Upregulation of P-JNK and P-p38 Signaling Pathways. Antibiotics.

[8] Moon S.H., Chung Y.C., Hyun C.G. (2019). Tobramycin Promotes Melanogenesis by Upregulating p38 MAPK Protein Phosphorylation in B16F10 Melanoma Cells. Antibiotics.

[9] Hyun S.B., Chung Y.C., Hyun C.G. (2020). Nojirimycin suppresses inflammation via regulation of NF-κ B signaling pathways. Pharmazie.

[10] Kang H.K., Hyun C.G. (2020). Anti-inflammatory Effect of d -(+)-Cycloserine Through Inhibition of NF-κB and MAPK Signaling Pathways in LPS-Induced RAW 264.7 Macrophages. Nat. Prod. Commun..

[11] Kang J.K., Kang H.K., Hyun C.G. (2022). Anti-inflammatory effects of spiramycin in LPS-activated RAW 264.7 macrophages. Molecules.

[12] Rastelli G., Pellati F., Pinzi L., Gamberini M.C. (2020). Repositioning Natural Products in Drug Discovery. Molecules.

[13] Atanasov A.G., Zotchev S.B., Dirsch V.M., Supuran C.T., International Natural Product Sciences Taskforce (2021). Natural products in drug discovery: Advances and opportunities. Nat. Rev. Drug Discov..

[14] Ribaudo G., Memo M., Gianoncelli A. (2021). A Perspective on Natural and Nature-Inspired Small Molecules Targeting Phosphodiesterase 9 (PDE9): Chances and Challenges against Neurodegeneration. Pharmaceuticals.

[15] Mazzini S., Musso L., Dallavalle S., Artali R. (2020). Putative SARS-CoV-2 Mpro Inhibitors from an In-House Library of Natural and Nature-Inspired Products: A Virtual Screening and Molecular Docking Study. Molecules.

[16] Chung Y.C., Hyun C.G. (2021). Inhibitory Effects of Pinostilbene on Adipogenesis in 3T3-L1 Adipocytes: A Study of Possible Mechanisms. Int. J. Mol. Sci..

[17] Chung Y.C., Hyun C.G. (2020). Inhibitory Effects of Pinostilbene Hydrate on Melanogenesis in B16F10 Melanoma Cells via ERK and p38 Signaling Pathways. Int. J. Mol. Sci..

[18] Chung Y.C., Kim S., Kim J.H., Lee G.S., Lee J.N., Lee N.H., Hyun C.G. (2017). Pratol, an O-Methylated Flavone, Induces Melanogenesis in B16F10 Melanoma Cells via p-p38 and p-JNK Upregulation. Molecules.

[19] Kim M.J., Kim S.S., Park K.J., An H.J., Choi Y.H., Lee N.H., Hyun C.G. (2016). Methyl jasmonate inhibits lipopolysaccharide-induced inflammatory cytokine production via mitogen-activated protein kinase and nuclear factor-κB pathways in RAW 264.7 cells. Pharmazie.

[20] Yoon W.J., Ham Y.M., Yoon H.S., Lee W.J., Lee N.H., Hyun C.G. (2013). Acanthoic acid inhibits melanogenesis through tyrosinase downregulation and melanogenic gene expression in B16 melanoma cells. Nat. Prod. Commun..

[21] Kim Y.J., Lee G.H., Kwong B.Y., Martires K.J. (2019). Evidence-based, Skin-directed Treatments for Cutaneous Chronic Graft-versus-host Disease. Cureus.

[22] Marka A., Carter J.B. (2020). Phototherapy for Cutaneous T-Cell Lymphoma. Dermatol. Clin..

[23] Carbone A., Montalbano A., Spanò V., Musante I., Galietta L.J.V., Barraja P. (2019). Furocoumarins as multi-target agents in the treatment of cystic fibrosis. Eur. J. Med. Chem..

[24] Buhimschi A.D., Gooden D.M., Jing H., Fels D.R., Hansen K.S., Beyer W.F., Dewhirst M.W., Walder H., Gasparro F.P. (2020). Psoralen Derivatives with Enhanced Potency. Photochem Photobiol..

[25] Rodrigues J.L., Rodrigues L.R. (2021). Biosynthesis and heterologous production of furanocoumarins: Perspectives and current challenges. Nat. Prod. Rep..

[26] Bonte F., Dumas M., Chaudagne C., Meybeck A. (1994). Influence of asiatic acid, madecassic acid, and asiaticoside on human collagen I synthesis. Planta Med..

[27] Maquart F.X., Bellon G., Gillery P., Wegrowski Y., Borel J.P. (1990). Stimulation of collagen synthesis in fibroblast cultures by a triterpene extracted from *Centella Asiatica*. Connect. Tissue Res..

[28] Lee J., Jung E., Park J., Jung K., Lee S., Hong S., Park J., Park E., Kim J., Park S. (2005). Anti-inflammatory effects of magnolol and honokiol are mediated through inhibition of the downstream pathway of MEKK-1 in NF-kappaB activation signaling. Planta Med..

[29] Park J., Lee J., Jung E., Park Y., Kim K., Park B., Jung K., Park E., Kim J., Park D. (2004). In vitro antibacterial and anti-inflammatory effects of honokiol and magnolol against *Propionibacterium* sp.. Eur. J. Pharmacol..

[30] Zhang H.L., Wu X.Y., Mi J., Peng Y.J., Wang Z.G., Liu Y., Wu X.L., Gao Y. (2017). A New Anti-Inflammatory Alkaloid from Roots of *Heracleum dissectum*. Chem. Biodivers..

[31] Kang J.K., Chung Y.C., Hyun C.G. (2021). Anti-Inflammatory Effects of 6-Methylcoumarin in LPS-Stimulated RAW 264.7 Macrophages via Regulation of MAPK and NF-κB Signaling Pathways. Molecules.

[32] Zhou C., Zhang X., Ruan C.C., Cheang W.S. (2021). Two methoxy derivatives of resveratrol, 3,3′,4,5′-tetramethoxy-trans-stilbene and 3,4′,5-trimethoxy-trans-stilbene, suppress lipopolysaccharide-induced inflammation through inactivation of MAPK and NF-κB pathways in RAW 264.7 cells. Chin. Med..

[33] Kim G.H., Cheong K.A., Lee A.Y. (2017). Increased Skin Irritation by Hydroquinone and Rsetinoic Acid Used in Combination. Ann. Dermatol..

[34] Hyun S.B., Bae S., Hyun C.G. (2020). Antioxidant Activities of Jeju Wax Apple (*Syzygium samarangense*) and Safety of Human Keratinocytes and Primary Skin Irritation Test. Cosmetics.

